# Graham-Little-Piccardi-Lassueur syndrome: A rare case associated with a chromosomal anomaly and unusual histopathologic characterization of keratosis pilaris-like lesions

**DOI:** 10.1016/j.jdcr.2025.09.019

**Published:** 2025-10-23

**Authors:** Timothy Klufas, Ayushya Ajmani, Albert Zhou, Campbell L. Stewart, Brett Sloan, Gillian Weston

**Affiliations:** a New York Medical College, School of Medicine, Valhalla, New York; b Department of Dermatology, University of Connecticut, Farmington, Connecticut; c Geisel School of Medicine at Dartmouth, Hanover, New Hampshire

**Keywords:** Alopecia, Chromosome 8 Duplication/Deletion, GLPLS, Graham-Little-Piccardi-Lassueur Syndrome, Keratosis PilarisLichen Planopilaris, LPP

## Introduction

Graham-Little-Piccardi-Lassueur syndrome (GLPLS) is a rare subtype of lichen planopilaris (LPP) primarily affecting middle-aged women.^1^^,^^2^ The condition is often characterized by the triad of fibrosing alopecia of the scalp, non-fibrosing alopecia of the axillae and groin, and follicular keratotic papules; however, not all cases present with this triad.^1^, ^2^, ^3^ Histopathologic features are rarely reported for GLPLS, with most diagnoses relying on clinical findings alone. We report a young male patient with a history of deletion/duplication on chromosome 8 and GLPLS, which highlights the possibility of a genetic predisposition associated with disease pathogenesis. We also add to the limited literature on the histopathologic features of keratosis pilaris-like papules and also report on follicular rupture—which has not been previously described.^4^^,^^5^ Finally, our case expands the discussion of emerging treatments such as JAK inhibitors and other hair regrowth therapies for scarring alopecias.

## Case report

**Fig 1:**
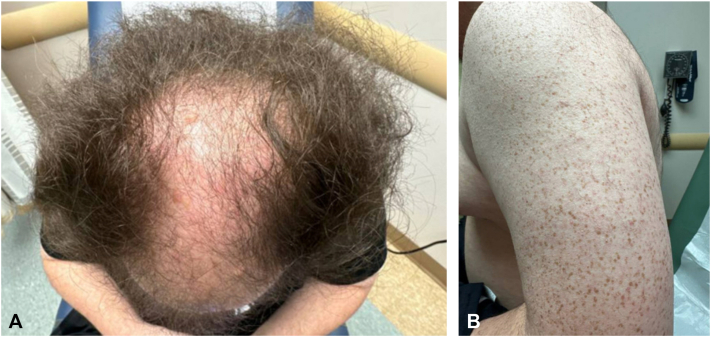
Diffuse patchy hair loss on the vertex, midline, and fronto-temporal scalp with perifollicular erythema **(A)**. Multiple follicular papules on left upper arm clinically reminiscent of keratosis pilaris **(B)**.

A 24-year-old male with a history of premature birth and a chromosomal deletion/duplication on chromosome 8 (invdupdel(8p)) presented to the clinic with alopecia. His family history was notable for androgenetic alopecia (AGA) in his father. The patient reported intermittent use of over-the-counter minoxidil 5% foam without improvement. He denied associated symptoms such as pruritus, erythema, or systemic signs of illness. On examination, the patient had significant hair loss affecting the vertex, midline, and fronto-temporal scalp with perifollicular erythema and scale; there was also mild thinning of the lateral eyebrows (Fig 1, *A*). Some evidence of mild alopecia in the proximal superior aspect of the right axilla was also observed. Initial differential diagnosis included AGA, alopecia areata, and lichen planopilaris (LPP). Additionally, numerous small, rough, follicular papules with sandpaper-like texture were noted on the bilateral upper extremities, features consistent with keratosis pilaris (KP) (Fig 1, *B*).

**Fig 2:**
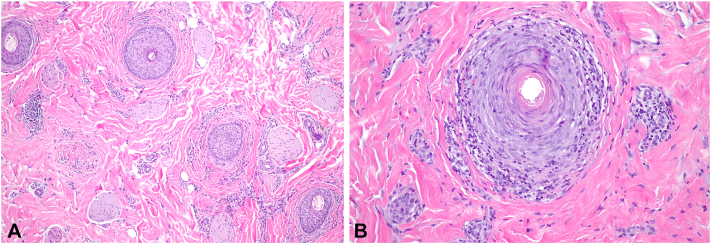
Punch biopsy of the scalp showing decreased follicular density **(A)** replacement of follicles by fibrous tracts. Perifollicular inflammation with dyskeratosis and epithelial atrophy and fibrosis affects the remaining follicles **(B)**.

**Fig 3:**
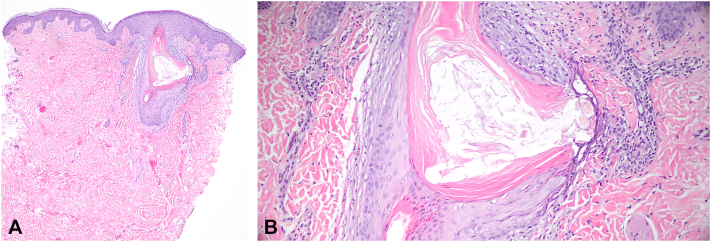
Punch biopsy of the right shoulder demonstrates a dilated follicle at the level of the infundibulum with a keratotic plug with surrounding perifollicular and perivasulcar inflammation **(A)**. The follicle is ruptured with extrusion of hair shafts into the adjacent superficial reticular dermis **(B)**.

A punch biopsy was performed on the scalp to further characterize the subtype of hair loss. Histopathology revealed lymphocyte-mediated scarring alopecia with diminished follicular density, perifollicular fibrosis, eccentric follicular atrophy and dyskeratosis, and absence of sebaceous glands (Fig 2). These features were consistent with LPP and taken together, with the thinned eyebrows and KP-like papules, the patient was diagnosed with GLPLS. In efforts to maximally characterize this patient’s presentation, a biopsy of a follicular papule on the right shoulder was also performed and revealed a keratotic plug at the follicular infundibulum with follicular rupture and extrusion of hair shafts into the superficial dermis (Fig 3).

Treatment was initiated with topical clobetasol 0.5% solution alternating with topical ruxolitinib 1.5% cream, intralesional steroid injections, oral hydroxychloroquine 200 mg twice daily, and oral minoxidil 2.5 mg. After 4 months of sustained treatment, the hair loss stabilized with diminished background inflammation, but minimal regrowth was noted. A genetics referral was made for further genomic characterization, and although a mutation related to alopecia was not identified, he was found to harbor a monoallelic pathogenic TNNT2 mutation associated with cardiomyopathy.

## Discussion

GLPLS remains a poorly understood subtype of LPP. It is classically characterized by a triad of patchy scarring alopecia of the scalp, non-scarring alopecia of the groin and axillae, and follicular keratotic papules; however, not all cases exhibit these features.^3^ The condition predominantly affects post-menopausal women, with most reported cases occurring in individuals aged 30 to 50 years. It is rarely documented in young men, making this 24-year-old male particularly unusual.^1^^,^^2^ Our case expands the demographic understanding of the condition and adds to the limited literature on the histopathologic characterization of these keratotic papules.^4^^,^^5^

Compared to previous reports that have identified enlarged perifollicular infundibulum with perifollicular lymphocytic infiltrate,^5^ this finding was not observed in our case. While KP-like papules are often clinically diagnosed in GLPLS, histopathologic characterization of them as in this patient are hardly reported. The observed follicular rupture and extrusion of hair shafts into the dermis may represent an unusual finding in the setting of GLPLS, suggesting an extension of immune-mediated injury to follicular structures, potentially linking KP-like changes to the broader inflammatory and fibrotic processes underlying the disease. This histologic finding may better define diagnostic criteria, and the need to integrate clinical and microscopic evaluation when GLPLS is suspected.

Genetic studies have linked central centrifugal cicatricial alopecia (CCCA) and frontal fibrosing alopecia (FFA) to specific gene mutations, including PADI3 mutations on chromosome 1 and ERAP1/MHC class I variants on chromosome 5, respecitvely.^6^ Given the clinical and histopathological similarities between FFA and GLPLS, it is plausible that chromosome 8p alterations may represent a potential, yet undefined, locus for LPP-related disorders. This chromosomal abnormality has been associated with a spectrum of neurodevelopmental disorders, subtle facial dysmorphisms, and alopecic changes, including frontal balding.^7^ Specifically, invdupdel(8p) has been implicated in immune dysregulation, including alterations in pathways such as interferon-gamma and JAK-STAT signaling, which also are important in the pathogenesis of LPP and its subtypes.^8^ While a whole genome study was inconclusive, a pathogenic variant might not yet have been identified. Nevertheless, there may be a potential link between this particular chromosomal aberration and the onset of inflammatory alopecias and although the patient did not exhibit cognitive impairments or overt dysmorphic features, his clinical presentation may represent a phenotypic, multifactorial, or epigenetic continuum of this genetic anomaly.

Management of GLPLS remains challenging due to its chronic, progressive nature and the limited efficacy of therapies in reversing follicular damage. Early intervention is essential to prevent irreversible scarring. In this case, disease progression was halted with a multifaceted anti-inflammatory regimen, and oral minoxidil was added adjunctively, however, significant regrowth has not been observed.^9^ Emerging therapies, including JAK inhibitors, PPARγ agonists (eg, pioglitazone), and metformin, show promise in modulating immune and inflammatory pathways.^10^ Other treatments such as platelet-rich plasma (PRP), low-level laser therapy, and hair transplantation may also be beneficial.^10^

This case broadens the phenotypic and histopathologic understanding of a unique scarring alopecia subtype and highlights a potential genetic link through chromosome 8p anomalies. Moreover, the potential epigenetic and immunologic contributions of invdupdel(8p) provide a foundation for future research into the pathogenesis and clinical spectrum of scarring alopecias.

## Conflicts of interest

None disclosed.
